# Editorial: Mechanism study of bioactive molecules using omics technology

**DOI:** 10.3389/fcell.2025.1720475

**Published:** 2025-10-22

**Authors:** Linhui Zhai, Bingbing Hao, Eric B. Dammer, Zhongwei Xu

**Affiliations:** ^1^ Translational Research Institute of Brain and Brain-Like Intelligence, Shanghai Fourth People’s Hospital, School of Medicine, Tongji University, Shanghai, China; ^2^ Tianjian Laboratory of Advanced Biomedical Sciences, Academy of Medical Sciences, Zhengzhou University, Zhengzhou, China; ^3^ Department of Neurology, Emory University School of Medicine, Atlanta, GA, United States; ^4^ Central Laboratory, Logistics University of Chinese People’s Armed Police Force, Tianjin, China

**Keywords:** genomics, transcriptomics, proteomics, metabolomics, drug target, mechanism, bioactive molecule

## Introduction

This Frontiers Research Topic aims to emphasize the role of omics technologies in elucidating the mechanisms of bioactive molecules. Bioactive molecules—ranging from plant-derived phytochemicals and microbial metabolites to endogenous peptides and synthetic small molecules—hold immense potential for advancing human health, agriculture, and environmental sustainability. However, their full therapeutic and functional potential remains largely unrealized due to the complexity of their mechanisms of action. These molecules often engage multiple biological targets, modulate interconnected signaling pathways, and produce context-dependent effects that vary across cell types and organisms. For decades, conventional experimental approaches—such as single-target assays and candidate-gene studies—have yielded only fragmented insights into these complex interactions (Hopkins 2008), thereby constraining efforts to rationally optimize bioactive molecules for applications in medicine, crop protection, and bioremediation.

## Omics unveils comprehensive mechanisms of natural compounds

In recent years, the emergence of omics technologies—high-throughput methodologies that enable comprehensive profiling of genomes, transcriptomes, proteomes, metabolomes, and other molecular layers—has profoundly transformed this field. By capturing system-wide biological responses to bioactive compounds, omics approaches effectively bridge the gap between observed phenotypic outcomes and their underlying molecular mechanisms, thereby advancing mechanism-driven research into new frontiers.

The study of bioactive molecules has traditionally relied on a reductionist approach—identifying a single molecular target, such as a receptor or enzyme, and examining how a compound binds to or inhibits it. However, this paradigm fails to capture the broader “network effect” exhibited by bioactive molecules. For instance, plant polyphenols like resveratrol do not act exclusively on sirtuins. They modulate hundreds of proteins, alter metabolite profiles, and reprogram gene expression networks associated with inflammation, oxidative stress, and aging.

Omics technologies overcome this limitation by offering a comprehensive, system-level perspective on biological changes (Yan et al. 2015). Transcriptomics, for example, can uncover how a bioactive compound reprograms gene expression in specific cell lines or tissues, thereby revealing previously unrecognized functional pathways. Proteomics complements these insights by detecting post-translational modifications—such as phosphorylation and acetylation—that modulate protein activity, while metabolomics captures dynamic alterations in small-molecule metabolites, providing a direct measure of biochemical pathway disruption. Collectively, these approaches convert isolated observations into robust, data-driven hypotheses regarding molecular mechanisms.

A recent study on curcumin, a polyphenol with anti-inflammatory properties, used integrative multi-omics—combining transcriptomic, proteomic, and metabolomic data from human immune cells—to identify NF-κB, a key inflammatory transcription factor, as central to curcumin’s action (El-Saadony et al. 2025). Experiments showed that curcumin inhibits NF-κB by stabilizing its inhibitor, IκBα, demonstrating how multi-omics turns big data into actionable mechanistic insights. Multi-omics has also clarified the mechanisms of traditional Chinese medicine (TCM) compounds, which often act on multiple targets. For example, puerarin, a flavonoid from Pueraria lobata, was found to directly bind the GABAA receptor α1 subunit (GABRA1) using photoaffinity chemical proteomics (Liu et al. 2024). Cryo-EM revealed the binding site, showing that puerarin inhibits dorsal motor nucleus (DMV) neurons and reduces intestinal fat absorption, supporting its anti-obesity potential. Similarly, artemisinin, a sesquiterpene lactone with antimalarial activity, was shown via immunoprecipitation-mass spectrometry (IP-MS) to target LONP1 (lon protease 1) (Jeon et al. 2020). By enhancing the LONP1-CYP11A1 interaction, artemisinin reduces ovarian androgen synthesis, offering a new therapy for polycystic ovary syndrome (PCOS). These studies illustrate how omics advances our understanding of molecular mechanisms. Our frontiers Research Topic this Research Topic presents recent advances in the mechanistic research of various active molecules utilizing omics technologies, encompassing the following relevant topics.

Liquiritigenin is a planar dihydroflavonone monomer with strong anti-inflammatory activity. Liu et al. used super-SILAC to investigate the anti-inflammatory effects and epigenetic mechanisms of liquiritigenin (Liu P et al. 2025). They profiled histone post-translational modifications (PTMs) in M1 macrophages after treatment and identified key PTM sites modulated by liquiritigenin. Integrated transcriptome analysis showed that it exerts anti-inflammatory effects by altering histone PTMs and regulating gene expression via the PPAR signaling pathway.

Nervonic acid (NA) shows therapeutic potential in neurological disorders. Li et al. investigated the therapeutic effects of NA on cerebral ischemia-reperfusion injury in a rat model using whole-blood proteomic analysis (Li K et al., 2025). Compared to ginkgo biloba extract (EGb), NA primarily influenced proteins involved in oxidative stress response and calcium-dependent adhesion. Key targets of NA in middle cerebral artery occlusion (MCAO) models were ENO1, STAT3, NME2, VCL, and CCT3.

Geniposidic acid (GPA) has hypoglycemic, hypolipidemic, and choleretic effects. Tang et al. combined metabolomics and network pharmacology to investigate GPA’s anti-hyperlipidemic effect in high-fat diet-induced mice (Tang et al., 2025). They revealed that both GPA and lovastatin normalized metabolic disturbances, particularly in the tricarboxylic acid (TCA) cycle, glycolysis, amino acid metabolism, and ketone body synthesis. Molecular docking revealed strong binding between GPA and key proteins—including ALB, CAT, ACACA, ACHE, and SOD1—indicating their potential as therapeutic targets.

Sinomenine is a key bioactive alkaloid from Sinomenium acutum Li et al. used plasma metabolomics to identify sinomenine-induced changes in amino acid metabolism and 94 potential plasma biomarkers associated with pathways such as valine, leucine, isoleucine biosynthesis, and glycine, serine, threonine metabolism (Li Q et al., 2025). Molecular docking and SPR confirmed that sinomenine specifically targets glutamine synthetase, inhibiting its activity and GLUL expression, thereby reducing glutamine levels in RA-FLS.

Irisin promotes bone mass recovery and maintains bone health. Xing et al. used transcriptomics and molecular biology experiments to confirm that irisin reshapes bone metabolic homeostasis and delays age-related osteoporosis by regulating BMSC differentiation via the Wnt signaling pathway (Xing et al. 2024).

Chemical probes offer new tools for compound target identification. Liu et al. developed a phenol thiol probe designed to mimic cysteine, which reacts with Mollugin and related natural products via electrophilic free radical addition, incorporating both the probe and a hydroxyl group into the adducts (Liu T et al., 2025).

## Challenges and innovations

Despite its significant technological advantages, omics-driven mechanistic research faces several challenges. First, data quality and standardization remain major challenges: differences in sample preparation, sequencing platforms, and analysis pipelines affect reproducibility. Second, omics findings require rigorous validation, as high-throughput methods often yield false positives; mechanistic hypotheses must be confirmed with targeted low-throughput experiments—such as CRISPR knockout or *in vitro* binding assays. Third, the high cost and limited access to omics technologies restrict their use in low-resource settings, where many bioactive compounds, including those from traditional medicinal plants, are found. Lowering sequencing and metabolomics costs and strengthening capacity-building are essential to closing this gap.

Looking ahead, several key trends are poised to shape the future of this field. Single-cell omics will allow researchers to investigate the mechanisms of bioactive molecules at the cellular level, revealing differential responses among distinct cell types within a tissue to the same molecule. Spatial omics, which maps molecular alterations across tissue sections, will further enhance our understanding of context-dependent effects—for instance, how chemotherapeutics selectively target tumor cells while sparing adjacent healthy tissues. Moreover, multi-species omics will provide insights into inter-organismal interactions, such as how metabolites produced by gut microbiota influence host physiology or how plant-derived compounds affect insect pests. The next-generation of omics technologies is expected to address current limitations. For example, peptide-centric local stability assay (PELSA) (Li G et al., 2025) and limited proteolysis coupled with mass spectrometry (LiP-MS) (Feng et al., 2014) enable label-free identification of molecular targets by detecting ligand-induced protein conformational changes, eliminating the need for probe design and thereby extending its utility to non-covalent binders.

## Conclusion

The study of bioactive molecule mechanisms has entered a new era thanks to omics technology. Researchers can now move beyond single targets or isolated pathways to explore the full complexity of biological systems, revealing hidden interactions and context-dependent effects. By integrating multi-omics data with advanced computational tools, scientists are turning big data into actionable insights that accelerate translation from bench to bedside, field, and environment. However, challenges related to standardization and accessibility persist, necessitating coordinated efforts across the scientific community. As these technologies advance, omics approaches will enhance our understanding of bioactive molecules and enable their potential to address critical global issues—such as disease, food security, and environmental degradation. They are poised to fully realize the therapeutic potential of bioactive compounds, thereby accelerating the development of precision medicine.
